# Combined Antimicrobial Activity of Photodynamic Inactivation and Antimicrobials–State of the Art

**DOI:** 10.3389/fmicb.2018.00930

**Published:** 2018-05-08

**Authors:** Agata Wozniak, Mariusz Grinholc

**Affiliations:** Laboratory of Molecular Diagnostics, Department of Biotechnology, Intercollegiate Faculty of Biotechnology, University of Gdansk and Medical University of Gdansk, Gdansk, Poland

**Keywords:** antimicrobials, antimicrobial photodynamic inactivation, photoinactivation, photosensitizers, synergy

## Abstract

Antimicrobial photodynamic inactivation (aPDI) is a promising tool for the eradication of life-threatening pathogens with different profiles of resistance. This study presents the state-of-the-art published studies that have been dedicated to analyzing the bactericidal effects of combining aPDI and routinely applied antibiotics in *in vitro* (using biofilm and planktonic cultures) and *in vivo* experiments. Furthermore, the current paper reviews the methodology used to obtain the published data that describes the synergy between these antimicrobial approaches. The authors are convinced that even though the combined efficacy of aPDI and antimicrobials could be investigated with the wide range of methods, the use of a unified experimental methodology that is in agreement with antimicrobial susceptibility testing (AST) is required to investigate possible synergistic cooperation between aPDI and antimicrobials. Conclusions concerning the possible synergistic activity between the two treatments can be drawn only when appropriate assays are employed. It must be noticed that some of the described papers were just aimed at determination if combined treatments exert enhanced antibacterial outcome, without following the standard methodology to evaluate the synergistic effect, but in most of them (18 out of 27) authors indicated the existence of synergy between described antibacterial approaches. In general, the increase in bacterial inactivation was observed when both therapies were used in combination.

## Introduction

Almost 89 years have passed since Alexander Fleming discovered penicillin–the antibiotic that revolutionized medicine–and contributed to research associated with the golden age of antibiotics (Davies, 2006; Tan and Tatsumura, 2015). Microbiologists and clinicians are currently struggling with the increasing frequency of drug resistance among pathogenic bacteria (Nakonechny and Nisnevitch, 2011). According to the antimicrobial resistance report published in 2016, the number of deaths caused each year by pathogenic bacteria will increase to 10 million by 2050 if no actions are taken (O'Neill, 2016); scientists are thus now focused on finding new biocidal substances or methods to effectively cope with emerging drug resistance. A few of the most recent examples include (i) the discovery of a new antibiotic by researchers at Rutgers University–pseudouridimycin, produced by microorganisms isolated from soil (Maffioli et al., 2017), (ii) the acquisition of a new class of antibiotics (Teixobactin) from the soil bacterium *Eleftheria terrae* (Fiers et al., 2017), and (iii) the discovery of the cathelicidins by researchers at Sydney University-these antimicrobial peptides are acquired from Tasmanian devil and active against gram-positive and gram-negative bacteria (Peel et al., 2016). These new compounds effectively fight against drug-resistant bacteria. However, the problem of rapidly growing resistance is still present and unsolved. Scientists engaging this problem should focus on alternative approaches to eradicating pathogenic bacteria (Wainwright et al., 2016). Antimicrobial photodynamic inactivation (aPDI), also known as a photodynamic antimicrobial therapy (PACT) and photodynamic inactivation (PDI), is an alternative method to fight resistant microorganisms, including bacteria, fungi, parasites and viruses (Awad et al., 2016; Hamblin, 2016). The aPDI method requires the presence of oxygen, a non-toxic photosensitizer (PS) and light. The PS is activated by the absorption of a photon with a specific wavelength, and this absorption leads to the formation of short-lived excited states of the PS. These states are then transformed to a triplet excited state, which further progresses along two separate photochemical pathways. In a type 1 mechanism, an electron is transferred from the triplet state of the PS and promotes the creation of reactive oxygen species (ROS), e.g., hydroxyl radicals (HO^·^). In a type 2 mechanism, the energy from the triplet state of the PS is transferred to produce singlet oxygen radicals (^1^O_2_). These compounds promote oxidative stress, which results in DNA damage and the destruction of cell envelopes, lipids and other components whose dysfunction finally leads to cell death. Moreover, aPDI confers numerous positive effects. The main advantage of aPDI is that bacterial resistance does not develop as a result of the treatment, which is due to the production of widely acting and indirectly targeted ROS during aPDI (Denis and Hamblin, 2011). Next, aPDI could affect the activity and/or production of numerous virulence factors, leading to decreased bacterial pathogenicity unlike antibiotic therapy, which can promote the production of virulence factors and lead to an increase in their release upon antibiotic treatment (Kharkwal et al., 2012; Fila et al., 2016; Dai, 2017; Wang et al., 2017). Furthermore, the aPDI is not cyto- and phototoxic toward eukaryotic cells in a wide therapeutic window and does not promote mutagenic effects in treated eukaryotic and prokaryotic cells (Grinholc et al., 2015).

The aPDI method has been repeatedly demonstrated in the literature to have many more advantages than individual routine antibiotic therapies. First, this method functions in a short time and limited space, potentially inactivating only the microorganisms that are present in the infection site without negatively influencing physiological flora (Ryskova et al., 2010). Second, the literature does not show that aPDI leads to the development of resistance against aPDI. Numerous studies have shown that habituation or incubation of bacterial cells with sublethal aPDI doses does not result in the development of resistance against phototreatments (Cassidy et al., 2010; Tavares et al., 2010). The main targets for aPDI are various structures and components of bacterial cells instead of one major target (as in the case of antibiotics), which reduces the possibility of developing resistance against such approaches (Maisch, 2015). Finally, the biocidal factors in aPDI are ROS and singlet oxygen; mechanisms of resistance against these species have not been discovered. Another unquestionable advantage of aPDI is its effectiveness in the inactivation of numerous virulence factors (Fila et al., 2016). The aPDI method may decrease the activity of proteases, lipases, secreted toxins, etc., (Fila et al., 2016).

In most research in this area, aPDI has been proposed as an alternative treatment option that acts independently and in isolation from complementary antimicrobial approaches, e.g., antibiotic therapy. Use of aPDI alone should lead to the successful eradication of pathogenic microorganisms from the site of infection. However, the achievement of satisfactory clinical effects with photodynamic inactivation, understood as the total eradication of microorganisms at the site of infection, is extremely difficult and rarely described despite numerous works carried out worldwide. Two important limitations of the aPDI method are its lower bactericidal efficacy against microorganisms growing in biofilms and the fact that the efficiency it exhibits in *in vitro* studies rarely translates into animal models. Even after the effective elimination of microorganisms from the site of infection, the regrowth of microorganisms and recurring development of the infection are observed 24 h post-treatment. Nevertheless, we are deeply convinced that photoinactivation has many advantages that make it an attractive tool for a comprehensive fight against multiresistant human pathogens. We therefore suggest the use of the unquestionable advantages of photodynamic inactivation to sensitize multidrug-resistant microorganisms to chemotherapeutic agents by pairing it with routinely used antibiotics. This approach allows the use of antimicrobial agents to which bacteria express high resistance and leads to significant decreases in the MIC, enabling the eradication of microorganisms and inhibiting the regrowth of microbes at the infection site.

The most recent discovery concerning combined aPDI and antibiotics indicate that photoinactivation renders microbes susceptible for routinely used antimicrobials (Fila et al., 2016). If this phenomenon is confirmed using appropriate methodology and translated into *in vivo* and clinic applications, this approach could significantly reduce the rate of emerging drug resistance among pathogens due to the reduced use of antimicrobials employed in the treatment. Reviewing existing publications and searching for evidence-based proof of synergism between aPDI and antimicrobial activities are thus important, as is using appropriate experimental approaches for studying the synergy between these two treatments.

## Approved methodology for synergy testing

According to the *American Society for Microbiology*, only a few experimental procedures are adequate for determining synergistic effects between various antibacterial approaches (http://www.aac.asm.org). These methods include using (i) disk-diffusion assays, (ii) *E*-tests for antibiotic susceptibility testing, (iii) checkerboard assays, (iv) post-antibiotic effects (PAEs), and (v) the Bliss model for synergy testing in biofilm cultures (Habash et al., 2014).

### Disk-diffusion assay

This technique is a simple approach to test antimicrobial susceptibility in routine clinical microbiology laboratories (Matuschek et al., 2014). This assay operates by the diffusion of an antimicrobial agent from a disk to solid medium (typically M-H medium), which leads to the formation of circular zones of growth inhibition (Kuper et al., 2012). According to the EUCAST (European Committee On Antimicrobial Susceptibility Testing) guidelines, the disk-diffusion methodology includes the use of overnight bacterial inocula or colonies that are suspended in saline to obtain bacterial suspensions with an optical density of 0.5 McFarland (Matuschek et al., 2014). Bacterial suspensions are placed on M-H plates at some point from 15 to 60 min after preparation. The disks are then placed on M-H medium 15 min after incubation, and the incubation of antibiograms is performed at 35 ± 1°C for 16-20 h. The measurements of growth inhibition zones and interpretation of results are based on the EUCAST breakpoint tables and additional instructions (http://www.eucast.org). AST guidelines provide no indication about the change in inhibition zone value that justifies considering synergy, thus, any statistically significant change in growth inhibition zone could potentially indicate synergy.

### *E*-test

The *E*-test, known also as the epsilometer test, is also based on the diffusion of an antimicrobial agent in culture medium, but in contrast to disk-diffusion assays, this quantitative technique can be used to estimate MIC values. As in disk-diffusion assays, this method typically uses M-H medium and appropriate incubation conditions (Kuper et al., 2012). MIC values are validated by identifying the intersecting areas of growth inhibition on *E*-test strip scales (Kuper et al., 2012). This method defines synergy as a ≥ 3 dilutions in MIC, additivity as a decrease of ≥ 2 but < 3 dilutions and indifference as a decrease of < 2 dilutions in the MIC. Antagonism is defined as an increase of ≥ 3 dilutions of the MIC.

### Checkerboard assay

This method is often used to determine the interaction between and potency of two or even three factors. Serial 2-fold dilutions of tested compounds are prepared in 2-dimensional fashion in one microtiter plate(Jenkins and Schuetz, 2012). The type of interaction is determined based on the assessment of the fractional inhibitory concentration index (FICI) for each tested antimicrobial agent (FIC_A_, FIC_B_) (Rybak et al., 2014). However, these values are appointed for those concentrations of compounds which administered together lead to the inhibition of the bacterial growth; next, these values are compared with the MIC values for each agent tested separately (Doern, 2014). Thus, the determination of interaction is based on FICI value which is calculated as follow: ∑ FICI = FIC_A_ + FIC_B_, where FIC_A_ equals the MIC of drug A in combination divided by the MIC of drug A alone and FIC_B_ equals the MIC of drug B in combination divided by the MIC of drug B alone (Jenkins and Schuetz, 2012). The most recent guidelines given by the British Society for Antimicrobial Chemotherapy concerning checkerboard assays stated that based on this assay, one could determine the two following interactions: (i) synergy (when FICI is ≤ 0.5) and (ii) antagonism (when FICI is > 4.0). No other interactions, such as indifference, are defined by this method (Odds, 2003).

### Time-kill assay (TKA)

TKAs are performed in large volumes (>10 ml) in glass beakers where the bacterial inoculum is placed into broth that contains the desired concentration of antimicrobials. The inoculum is then incubated for 48 h, and 0.5 ml aliquots are periodically collected and plated for colony count determinations. These samplings generally occur at 4, 8, 10, 12, and 24 h. The time-kill colony counts are then graphically represented as a function of time. Synergy occurs in time-kill assays when the results of the antimicrobial combination are > 2 log_10_ greater than the results of the combination's most active constituent (Boluki et al., 2017).

### Post-antibiotic effect (PAE)

PAEs are defined as delays in bacterial regrowth after a brief exposure to an antibiotic at a specific concentration (MIC). A bacterial inoculum is exposed to multiple MIC dilutions of antibiotics that are later removed or inactivated. Next, the regrowth of bacterial cells resuspended in antibiotic-free medium is monitored every 0.5–2 h. The post-antibiotic effect is defined based on the following formula: *PAE* = *T*−*C*, where *T* is the estimated time for a bacterial culture population to increase by 1 log_10_ of viable cells from the number of bacterial cells that were present after the chemotherapeutic agent had been removed and *C* is the time of growth of untreated control cells. The difference in the time that a microorganism requires after contact with an antibiotic to increase its number of viable cells 10-fold compared to the time that untreated bacteria require is described as the PAE. This effect can depend on several factors: the bacterial species, antibiotic concentrations and the time of exposure of bacterial cells to chemotherapeutical agents (Odenholt, 2001).

### Bliss model

The following formula is used for the Bliss model: S = (f_X0_/f_00_) (f_0Y_/f_00_)-(f_XY_/f_00_), where f_XY_ refers to the biofilm biomass in the presence of the combined treatment at concentration X for chemotherapeutic A and concentration Y for chemotherapeutic B, f_X0_ and f_0Y_ refer to the biofilm biomass in the presence of the individual treatments at concentrations of X and Y, respectively, f_00_ refers to the biofilm biomass in the absence of treatments, and S corresponds to the degree of synergy. Positive values of S reflect synergy, while a negative value of S reflects an antagonistic interaction. This methodology was used successfully for investigating the influence of combined factors on *Pseudomonas aeruginosa* biofilms with the application of aPDI (Habash et al., 2014).

Only 5 of 27 studies reviewed within the current paper and concerning combined aPDI and antimicrobial treatments were performed in accordance with the approved methodology. Moreover, the most recent guidelines indicate that the synergy can be concluded when it is defined by use of two or more of the abovementioned methods. None of the available published studies meet these requirements.

## Other methodology used for synergy and/or combined effect testing

Unfortunately, most of the studies describing the use of combined aPDI/antimicrobial treatments were not designed in accordance with approved standards. The potential synergistic interactions between aPDI and antimicrobials were reported after the use of custom-made methodology.

The most often used method for defining synergy and/or combined effect is a serial dilution of bacterial suspensions irradiated in the presence of antibiotics at different concentrations and the subsequent calculation of the number of CFU/ml. A reduction in the viable counts of bacterial cells of 6 or more log_10_ defines a synergistic interaction (Pérez-Laguna et al., 2017). The same method was used by the Cassidy group, who described a synergistic effect as a reduction in viable counts by ≥ 2 log_10_ more than the reduction in counts by the most active single agent (Cassidy et al., 2012). The same methodology was employed by Ronqui et al. (2016), who stated that a synergistic effect was present in a combined aPDI and antibiotic treatment against biofilm cultures based on comparing the level of reduction of bacterial cells receiving monotherapy (aPDI) to the reduction level obtained using the combined treatment. A difference in reduction of 0.6 log_10_ between these two groups was defined to represent a synergistic effect.

It must be stated that all the studies testing the efficiency of the combined therapy to inactivate bacteria and/or detecting the synergic effect of both therapies are highly appreciated. It must be noted that some of described papers were just aimed at determination whether the combined treatments exert enhanced antibacterial outcome with no interest in synergy testing, but most of them indicated the existence of synergy between described antibacterial approaches. However, omitting what was the purpose of performed researches, it is worth to underline that if aPDI is expected to gain the attention of international microbiologists and clinicians communities, it must be tested with the standard and approved methodology. One should be aware that even if the antimicrobials and aPDI reveal enhanced bacterial killing when acting together, the results will only be reliable and convincing if they are confirmed with the employment of approved standards.

## Antimicrobial photodynamic inactivation combined with antimicrobials

### *In vitro* studies: planktonic cultures

#### Endogenously produced porphyrins

Microorganisms, due to the presence of haem synthesis pathway, are able to produce and accumulate endogenous porphyrins. Their production could be increased with the administration of the appropriate precursor, i.e., delta-aminolevulinic acid (ALA). These endogenously produced porphyrins could serve as a photosensitizer and were used repeatedly in eradication of numerous bacterial pathogens by inducing photochemical damages (Hamblin and Hasan, 2004; Grinholc et al., 2008). Reznick et al. (2013) published data in 2013 indicating that a combined treatment of visible light irradiation and gentamycin results in increased antibacterial effects against *Pseudomonas aeruginosa*. During their experiments, bacteria were irradiated with continuous or pulsed-switched light in the presence or absence of gentamicin for 24 h. Treating bacteria separately with gentamycin and green light (λ = 532 nm) in two exposure modes of irradiation did not reduce the number of viable counts (Reznick et al., 2013). The application of continuous or pulsed-switched light in combination with gentamycin for 24 h gave an 8 log_10_ greater reduction in viable counts than individual treatments (Reznick et al., 2013). Endogenous porphyrins were also used in eradication of *Clostridium difficile* which is an etiological agent of pseudomembranous colitis and is responsible for opportunistic infections in intensive care units, which are mainly caused by the eradication of natural flora as a result of antibiotic administration (Musher et al., 2013; De Sordi et al., 2015). Choi et al. (2015) proved that application of aPDI in combination with tetracycline (0.5 mg/ml) gave a 2 log_10_ increased reduction in viable count after 5 min of irradiation and 3 log_10_ after 10 min of light exposure. In addition, this effect could be further enhanced (4 log_10_ greater than the count reduction in the control group) when chitosan was applied (Choi et al., 2015). The application of aPDI in combination with tetracycline (1.0 mg/ml) or chloramphenicol reduced the number of viable counts for *C. difficile* by 7 log_10_ more than UVA monotherapy. Interestingly, Fila et al. (2016) proved in 2016 that the application of blue light (λ = 410 nm) with the presence of intracellular photosensitizing compound eradicates planktonic cultures of *P. aeruginosa* strains that presented multidrug resistance (MDR) and extensive drug resistance (XDR) profiles. Moreover, a combined application of sublethal dose of blue light (10 J/cm^2^) and tested antibiotics (gentamicin, meropenem, or ceftazidime) reduced the minimal inhibitory concentration (MIC) by 2- to 64-fold more than individual treatments. The synergistic effect of light and antimicrobial applications was estimated using a checkerboard assay, which is a reliable technique for testing synergetic or antagonistic interactions. This evidence was the first to indicate the synergistic effect of combining blue light and antibiotic treatments for *P. aeruginosa* (Fila et al., 2016). Another study of combining aPDI with antibiotics was presented by Pereira et al. (2017a) in 2017; this study proved that irradiation of *Escherichia coli* and *Staphylococcus aureus* with blue (470 nm) or red light (625 nm) for 10 min in the presence of ciprofloxacin (5 mg) is more effective than antibiotic monotherapy. Moreover, the presence of norfloxacin (10 mg) with blue or red irradiation also exerted the positive effect of combined treatment on *S. aureus*, displayed as an increase in the sizes of inhibition zones on antibiogram plates (Pereira et al., 2017a). An interesting case of combined aPDI/antimicrobial therapy was reported by Jeong et al. (2017), whose group used *Propionibacterium acnes* and erythromycin-loaded liposomes (OELL) in their experiments. Irradiation of bacterial cells with light (200 s) in the presence of liposomes containing erythromycin at a concentration of 1 μg/ml reduced the viable cell count by 1.99 log_10_ more than laser monotherapy (Jeong et al., 2017).

#### Exogenously administered porphyrin-based PS

An excellent example of the synergistic effect of combining light, exogenous photosensitizer and antimicrobial therapy on biofilms and planktonic cultures was presented by Iluz et al. (2018) in 2018. When the planktonic cultures of *S. aureus* were treated with deuteroporphyrin IX (DP) and oxacillin and irradiated with a light dose of 46 J/cm^2^, a synergistic interaction was observed for DP (2-9 μM) and oxacillin (MIC 250 μM) based on checkerboard assays. The synergistic effect with oxacillin (1 μg/ml) was also represented by changes in the survival rate of bacterial cells. Irradiation with a light dose of 46 J/cm^2^ with DP (17 μM) completely eradicated bacterial cells, and the synergistic effect was still present at lower concentrations of DP (4 μM), but the number by which the viable bacterial cell count was reduced was lower (~6 log_10_) than that observed with the higher dose of DP (Iluz et al., 2018). Interestingly, Iluz et al. (2018) verified how long the synergistic interaction for DP-aPDI treatments remains after their application and proved that the absence of light treatment and exposure to oxacillin leads to a smaller reduction in the number of bacterial cells. The application of aPDI in combination with oxacillin (4 μM) increased the reduction in the viable counts of planktonic cultures by 6 log_10_ over the reduction achieved by independent treatments. In 2013, Sana S. Dastgheyb presented results for this combined treatment against *E. coli, Staphylococcus epidermidis*, and a methicillin-resistant *S. aureus* (MRSA) clinical isolate. Compared to the treatment of bacteria with only antibiotics (vancomycin and ceftriaxone), which did not affect cell viability, the exposure of *S. aureus* to 5 h of irradiation in the presence of porphyrin *meso*-tetrakis(4-aminophenyl) porphyrin (TAPP) gave a 1-1.5 log_10_ reduction in viable counts. Combining aPDI treatment with ceftriaxone and vancomycin reduced the cell viability by a further 1-2 log_10_ from its value in control groups. Higher bactericidal effectiveness was obtained for tobramycin and chloramphenicol (2.5 and 1 log_10_ reductions in viable counts, respectively). The application of light and PSs to antibiotic treatments increased antibacterial efficacy by a further 2.5 log_10_ for tobramycin and 3 log_10_ for chloramphenicol over the efficacy in probes where only antibiotics were used. Furthermore, to investigate the type of interactions (synergism and antagonism) for tobramycin, chloramphenicol, PSs and light, a checkerboard assay was prepared for all tested strains. An *S. aureus* strain was used as a reference: when both antibiotics were used as a treatment, an additive effect was observed. The combination of light and both chemotherapeutics had also an additive effect for an *E. coli* reference strain. The synergistic effect of combined therapy was proven for *S. epidermidis* and the MRSA clinical isolate. The types of interactions were defined using the checkerboard assay and established by measuring FICI range (Dastgheyb et al., 2013). The application of aPDI in combination with different antibiotics (tobramycin, ceftriaxone, vancomycin, or chloramphenicol) increased the reduction in viable counts from 0.5 to 3 log_10_ over the reduction achieved with individual treatments. The effects of the synergistic interaction between treatment with light and antibiotics on multidrug resistant bacterial strains isolated from hospital wastewater and patients have been reported (Almeida et al., 2014). For all tested strains isolated from patients, irradiation with white light and *meso*-tetrakis(1-methylpyridinium)porphyrin (Tetra-Py^+^-Me) (5 μM) reduced the number of viable cells by 6-8 log_10_ after 270 min of exposure to light, while a significant reduction (by 4-5 log_10_) had already occurred after 90-180 min. In the case of strains isolated from hospital water probes containing the same species of microbial pathogens, the bactericidal effect (4 log_10_ reduction in viable counts) was observed after just 30 min of irradiation with Tetra-Py^+^-Me (5 μM). Moreover, adding ampicillin (32 μg/ml) to an *E. coli* suspension reduced the number of viable cells by a further 1 log_10_ after 180 min and 2 log_10_ after 270 min of irradiation from the number of viable cells irradiated without the presence of antibiotic. Adding chloramphenicol (32 μg/ml) and exposing the bacterial suspension to light and PS for 270 min reduced the number of viable cells by 2 log_10_ more than treatment with only light and Tetra-Py^+^-Me (Almeida et al., 2014). The positive effects of combining aPDI and antibiotic therapy *in vitro* and *ex vivo* were also reported by Branco et al. (2018) in 2018. In *in vitro* experiments, a reference *S. aureus* strain was irradiated with white light and Tetra-Py^+^-Me (5 μM) for 180 min with a variety of antibiotics: chloramphenicol (0.25 μM), kanamycin (2 μg/ml), penicillin G (0.125 μg/ml) and ampicillin (0.25, 0.5 and 1 μg/ml). After 180 min of irradiation, the number of viable cells was reduced by 8 log_10_ for all antibiotics (Branco et al., 2018). Compared with monotherapy with DP, the application of aPDI in combination with ampicillin at its highest concentration (1.0 μg/ml) improved the reduction in viable counts for planktonic cultures by 4 log_10_ (Branco et al., 2018).

#### Phenothiazines

The enhanced effectiveness of aPDI with phenothiazinium PS and antibiotics was also demonstrated by M.H. Shih and F.C. Huang in 2010 using *Mycobacterium fortuitum* in *in vitro* and *in vivo* experiments. Monotherapy (100 J/cm^2^, 50 μg/ml methylene blue, MB) resulted in reducing the number of colony forming units (CFUs) by 2-3 log_10_ from their number in untreated cells. A synergistic effect was obtained in *in vitro* studies when a light dose of 100 J/cm^2^ was applied with MB (50 μg/ml) after bacteria were incubated for 72 h with antimicrobial agents. The presence of antibiotics (amikacin, ciprofloxacin, or moxifloxacin) in respective concentrations of 0.5, 0.06 and 0.06 μg/ml reduced the mycobacterial cell viability by 2 log_10_ from its value in untreated cultures (Shih and Huang, 2011). The application of aPDI in combination with antibiotics in *in vitro* experiments improved the cell count reduction by a further 2 log_10_ over aPDI or antibiotic monotherapy (moxifloxacine). Another example of synergistic interactions between aPDI and antibiotic was presented by Ronqui et al. (2016) in 2016. The main subjects of their research were *S. aureus* and *E. coli*. Additionally, the appropriate treatment order for ciprofloxacin and red light with MB was verified in experiments. For *S. aureus*, when the application of ciprofloxacin (0.6 μg/ml) preceded irradiation with MB (50 μg/ml), the viable cell count reduction was ~5 log_10_. The same log_10_ reduction was reached for *E. coli* with an antibiotic concentration of 0.004 μg/ml and the same light dose (2.8 J/cm^2^) in comparison to *E. coli* receiving monotherapy with MB. However, when ciprofloxacin (0.004 μg/ml) was administered after light irradiation in the presence of MB (50 μg/ml), the viability of *S. aureus* cells was reduced by ~6 log_10_. No change in viable cell reduction was reported for *E. coli* upon different drug administration. The application of aPDI in combination with ciprofloxacin thus improved the reduction in viable counts for *E. coli* and *S. aureus* by 5-6 log_10_ over the reduction from light monotherapy. Another interesting application of combining aPDI with MB and antibiotic treatment was described by Oppezzo and Forte Giacobone (2017) in 2017. They used aPDI with antibiotic against persistent bacteria. Persistent microorganisms can survive the lethal effects of antibiotic treatments as a result of reversible and temporary phenotypic alteration (Oppezzo and Forte Giacobone, 2017). A *P. aeruginosa* strain was treated for as long as 180 min with visible light in the presence of a PS (MB, 15 μM), and ofloxacin was added immediately thereafter to the inoculum. The same experiment was conducted against persistent cells that tolerate ofloxacin. The antibiotic was first added to the same final concentration, and after 50 min of incubation, MB was administered. Exposure to light was initiated at minute 60 of the experiment and lasted up to 240 min. The survival fraction when ofloxacin was added at the beginning of the experiment was significantly lower at 240 min than tests when the antibiotic was added at 90 min, clearly indicating that the chemotherapeutic agent exerted a greater effect when combined with aPDI, even when treated cells were tolerant to the agent, i.e., in the case of persistent cells of *P. aeruginosa* (Oppezzo and Forte Giacobone, 2017). The application of aPDI in combination with antibiotic on persistent bacteria that tolerate ofloxacin reduced the viable counts by 6 log_10_ more than monotherapy (antibiotic treatment). The first literature evidence of a combined aPDI/antibiotics treatment against pandrug-resistant *Acinetobacter baumannii* was presented by Boluki et al. (2017) in 2017. The presented research also aimed at studying whether aPDI affects the level of expression of *pmrA* and *pmrB* genes, which are responsible for *Acinetobacter* resistance to colistin. The author stated that the exposure of *A. baumannii* to Toluidine Blue O (TBO) (50 mg/l) and light-emitting diode (LED) light for 60 and 90 s resulted in increased bacterial drug susceptibility, which was evidenced by disk-diffusion antibiograms using colistin, ceftazidime, piperacillin and doripenem. An aPDI treatment was also successful with regard to the expression of two genes responsible for colistin resistance. The expression of *pmrA* and *pmrB* was 6.1- and 4.9-fold lower, respectively, in cells treated with aPDI with TBO (0.37 mg/ml) and light (180 J/cm^2^) than in untreated cells, indicating that aPDI influenced the expression of genes responsible for the production of lipid A (a constituent of lipopolysaccharide, LPS) which is strictly linked with resistance to colistin (Boluki et al., 2017). These results may suggest a mechanism underlying the synergy between antimicrobials and light therapy. Most experiments in the literature have been performed with *S. aureus*, which is the main etiological agent responsible for nosocomial, mucosal and cutaneous infections (Navidinia, 2016; Pérez-Laguna et al., 2017). Multidrug-resistant strains, i.e., methicillin-resistant *S. aureus* (MRSA), play a major role in life-threatening infections (Orrett and Land, 2006).

#### Rose bengal (RB)

In 2017, Pérez-Laguna et al. (2017) published data presenting the bactericidal effectiveness of aPDI used in conjunction with mupirocin and linezolid on a reference *S. aureus* strain. Irradiation of planktonic cultures was performed using two light sources (LEDs and white metal halide (WMH) lamps) with rose bengal (RB) or methylene blue as PSs. Irradiation with the LED light (18 and 37 J/cm^2^) and the WMH lamp (37 J/cm^2^) was performed with tested antibiotics at two concentrations (1 and 10 μg/ml). Complete eradication was observed in all experiments but for different concentrations of PSs in combination with mupirocin and linezolid. The most pronounced results were obtained when the concentration of antibiotics was 10 μg/ml (Pérez-Laguna et al., 2017). The application of aPDI in combination with mupirocin or linezolid improved cell viability reduction by a further 2-6. log_10_ over the reduction by aPDI and antibiotic monotherapy. The amount of increased reduction in the combined treatment depended on the fluence and light source (Pérez-Laguna et al., 2017) The most current report published in 2018 indicated that combined treatment of aPDI and gentamycin was effective against *S. aureus* biofilm and planktonic cultures (Pérez-Laguna et al., 2018). Light irradiation (18 J/cm^2^) of planktonic cultures administered with rose bengal (0.03 μg/ml) resulted in ~2 log_10_ reduction in viable cells whereas the combined treatment with the presence of antibiotic in two concentrations (1 and 10 μg/ml) reached the viability reduction by ~4 and 5 log_10_ units (Pérez-Laguna et al., 2018). Experiments conducted by Pérez-Laguna et al. (2018) proved that combined treatment was more effective in comparison to aPDI monotherapy. The first *in vitro* study that presented the influence of combining aPDI and antibiotics in planktonic cultures was described by Cahan et al. (2010) in 2010. The effectiveness of aPDI increased when conjugates of PS and antimicrobials were used, i.e., kanamycin and RB (RBLKAN) or 6-penicillic acid and RB (RBLPA) (Fiebelkorn et al., 2003; Cahan et al., 2010). Irradiation of *S. aureus* with red light (2 J/cm^2^) and treatment with RB gave only 1 log_10_ reduction in viable count, whereas the presence of the conjugates RBLKAN and RBLPA (0.078 μM) decreased the viable count by 7 and 5 log_10_, respectively from its value in cultures treated only with light and a PS (Cahan et al., 2010). When *E. coli* was treated with aPDI (16 J/cm^2^) and RB, the number of viable cells decreased by 3 log_10_; treatment with the RBLKAN conjugate (20 μM) decreased the viable cell count by 5 log_10_ further than monotherapy (aPDI) (Cahan et al., 2010). A combination of phototherapy with routinely applied antibiotics is a method leading to the complete eradication of this widespread pathogen. Cahan et al. (2010) proved the effectiveness of combining aPDI with antibiotics (which were administered as conjugates), improving the viable cell reduction by 5-8 log_10_ from reduction achieved with monotherapy with light and RB.

Most of the studies mentioned above reported enhanced bactericidal outcomes if combined aPDI/antimicrobial treatments were employed. Contradictory results have been reported only by Ramírez et al. (2015), who demonstrated antagonistic interactions when using this combined treatment against *A. baumannii*. The application of blue and white light resulted in growth inhibition zones on petri dishes with LB medium smaller than those of bacteria untreated with light. Similar effects were reported in the case of green light irradiation. This phenomenon was especially observed in the cases of two antibiotics: minocycline and tigecycline. Interestingly, inhibition zones did not change when red light was used. When another medium was used, e.g., Mueller-Hinton (M-H) or blood agar, inhibition zones for the tested antimicrobials did not differ between control and irradiated samples. The same conclusions concerning increased resistance to both antibiotics after irradiation with blue light were drawn when liquid LB medium was used. For example, MIC values changed from 0.125 to 128 μg/ml for *A. baumannii* A42 after treatment with minocycline and blue light. The mentioned investigation was performed using a few clinically important *Acinetobacter* species such as *A. radioresistens* (Ar181L), *A. nosocomialis, A. calcoaceticus*, and *A. soli* and *E. coli, Klebsiella pneumoniae* and *Enterobacter cloacae* and blue light irradiation affected antimicrobial susceptibility to minocycline and tigecycline (Ramírez et al., 2015). The application of aPDI in combination with minocycline increased the MIC for *A. baumannii* strains between 16 and 128-fold over its value resulting from individual treatments.

Table 1 summarizes the published results concerning the efficacy of treating planktonic cultures *in vitro* with a combination of aPDI and antimicrobials.

**Table 1:** Summary of combined aPDI/antimicrobial treatments against planktonic cultures - *in vitro* experiments.

**References**	**Species**	**Photosensitizer/compound**	**Source of light**	**Wavelength [nm]**	**Intensity [mW/cm^2^]**	**Antibiotics**	**Max. viability reduction in comparison to monotherapy (light)**	**Max. viability reduction in comparison to monotherapy (antibiotic)**	**Applied methodology for determination of combined effect**
Reznick et al., 2013	*P. aeruginosa*	–	Nd:YAG laser- continuous	532	100	Gentamycin	8 log_10_	7.5 log	Bacterial viability (CFU/ml)
			Nd: YAG laser-Pulsed-Q-switched	532	106		8 log_10_	7.5 log	
Choi et al., 2015	*C. difficile*		UVA lamp UV801KL	315-400	No data	Tetracycline	7 log_10_	8 log_10_	Bacterial viability (CFU/ml)
Ramírez et al., 2015	*A. baumannii*		9 LED emitting diodes (three-LED module strips emitting blue, green or red light)	No data	No data	Tigecycline minocycline	No reduction	No reduction	MIC determination
Fila et al., 2016	*P. aeruginosa* (XDR, MDR)		Single-emitter diode lamp	405	15.7	Gentamicin	No data	128 x MBC^a^ reduction	Checkerboard assay
						Meropenem	No data	4 x MBC reduction	
						Ceftazidime	No data	32 x MBC reduction	
Pereira et al., 2017a,b	*E. coli S. aureus*		LED	470 625	no data	Ciprofloxacin	No data	Inhibition zone increased (≥3 mm)	Disk diffusion assay
						Norfloxacin	No data	Inhibition zone increased (3 mm)	
Jeong et al., 2017	*P. acnes*		Fiber-Coupled Laser System	671	20	Erythromycin (loaded in liposomes)	1.99 log_10_	No data	Bacterial viability (CFU/ml)
Iluz et al., 2018	*S. aureus* (MRSA)	DP	Blue light tube TL 20 W/03 ES	360-460	No data	Oxacillin	~6 log_10_	~10 log_10_	Checkerboard assay
Shih and Huang, 2011	*M. fortuitum*	MB	Metal halogen lamp	560-780	100	Ciprofloxacin	~2 log_10_	~0.5 log_10_	Bacterial viability (CFU/ml)
						Moxifloxacin	~1.5 log_10_	~2 log_10_	
						Amikacin	~1 log_10_	~1 log_10_	
Ronqui et al., 2016	*S. aureus E. coli*		Red LED	660	No data	Ciprofloxacin	~5 log_10_	No data	Bacterial viability (CFU/ml)
							~6 log_10_	No data	
Oppezzo and Forte Giacobone, 2017	*P. aeruginosa*		LED	637	44	Ofloxacin	~3 log_10_	~6 log_10_	Bacterial viability (log N/N_0_)
Pérez-Laguna et al., 2017	*S. aureus*		LED	625 515 ± 10	7.0 5.8	Mupirocin linezolid	~3 log_10_	~6 log_10_	Bacterial viability (CFU/ml)
							~2 log_10_	~6 log_10_	
			White metal halide (WMH) lamp	420-700	90	Mupirocin linezolid	~5 log_10_	~6 log_10_	
							~4 log_10_	~6 log_10_	
	*S. aureus*	RB	LED	625 515 ± 10	7.0 5.8	Mupirocin linezolid	4.5 log_10_ 3 log_10_	~7 log_10_ ~7 log_10_	Bacterial viability (CFU/ml)
			White metal halide (WMH) lamp	420-700	90	Mupirocin linezolid	5.5 log_10_ 5.5 log_10_	~6.5 log_10_ ~7 log_10_	
Pérez-Laguna et al., 2018	*S. aureus*		Green LED-lamp	515 ± 10	5.8	Gentamycin	6 log_10_	~5.5 log_10_	Bacterial viability (CFU/ml)
			White metal halide (WMH) lamp	420-700	90	Gentamycin	6 log_10_	~5.5 log_10_	
Cahan et al., 2010	*S. aureus E. coli*	RB conjugate with kanamycin and 6-aminopenicillinic acid	3 x White luminescent lamps (18 W)	400-700	1.4-1.7	Kanamycin, aminopenicillanic acid	~7 log_10_^b^ ~5 log_10_^c^	~8 log_10_^b^ ~6 log_10_^c^	Bacterial viability (CFU/ml)
							~5 log_10_^b^	~6 log_10_^b^	
Dastgheyb et al., 2013	*S. aureus* (MRSA)	TAPP	Sylvania white light (100 W, 120 V)	No data	No data	Tobramycin	~3 log_10_	~2.5 log_10_	Checkerboard assay
						Chloramphenicol	~2 log_10_	~3 log_10_	
Boluki et al., 2017	*A. baumannii*	TBO	LED FotoSan 630 nm LAD	630	2.000-4.000	Piperacillin ceftazidime doripenem colistin	No data	No data	Disk diffusion assay
Almeida et al., 2014	*E. coli*	TMPyP	White light lamp (OSRAM 21)	380-770	4	Ampicillin	2 log_10_	~8 log_10_	Bacterial viability (CFU/ml)
						Chloramphenicol	2 log_10_	~8 log_10_	
Branco et al., 2018	*S. aureus*		OSRAM 21	380-700 400-800	4	Ampicillin	~8 log_10_	~7 log_10_	Bacterial viability (CFU/ml)
						Chloramphenicol	~8 log_10_	~8 log_10_	
						Tetracycline	~8 log_10_	~7.3 log_10_	
						Penicillin G	~8 log_10_	~8 log_10_	
						Kanamycin	~8 log_10_	~7.4 log_10_	

a *Minimal Bactericidal Concentration*.

b *Kanamycin conjugate*.

c *Penicillinic acid conjugate*.

### *In vitro* studies: biofilm cultures

Most of the microorganisms grow as biofilms in their natural habitats. These biological conglomerates consist of bacterial communities existing in a matrix composed of polysaccharides, lipids, proteins and extracellular DNA (Santajit and Indrawattana, 2016). This microenvironment constitutes a mechanical and biochemical protection from PSs or antibiotics at concentrations as high as 1,000 times those that affect planktonic cultures, challenging the treatment of infections (Fu et al., 2013; Abouelfetouh et al., 2016).

#### Endogenously produced porphyrins

The first published experimental data describing the inactivation of *S. aureus* biofilm cultures with two sources of light were reported by Krespi et al. (2011); they reported the application of two different lasers and ciprofloxacin accompanied by the presence of endogenous porphyrins. A shockwave (SW) laser was used for biofilm disruption, and a near-infrared (NIR) laser was used for the eradication of bacterial cells that persist in planktonic cultures. Irradiation of *S. aureus* biofilms with both lasers and treatment with ciprofloxacin (0.3 mg/L) after the planktonic bacteria had been rinsed reduced the viable cell count by 85%, while irradiation of the bacterial cultures before rinsing gave only 66% reduction under the same experimental conditions. Furthermore, when the SW laser was used with the addition of ciprofloxacin (0.3 mg/L), biofilm and planktonic cultures were reduced by 64%, whereas biofilm cultures (after rinsing the planktonic bacteria) were reduced by 81% from the control group value (Krespi et al., 2011). The combined treatment with ciprofloxacin and both lasers was effective against *S. aureus* biofilms, reducing the biofilm cell counts by 81% from its value for untreated cells and by 44% from its value for cells receiving monotherapy with ciprofloxacin (Krespi et al., 2011). In other studies, Barra et al. (2015) and Zhang et al. (2017) presented results of experiments which were conducted with the presence of delta- aminolevulinic acid and antibiotics against *S. aureus*. Barra et al. (2015) reported a synergistic effect from the use of a combined aPDI and gentamycin treatment against three representatives belonging to *Staphylococcus*. In this study, 5-aminolevulinic acid (5-ALA) was used as a precursor for endogenous porphyrin production. A quantitative analysis of biofilms demonstrated that exposure to 2 μg/ml gentamycin followed by light irradiation (500 J/cm^2^) resulted in total eradication of *Staphylococcus haemolyticus*; however, the same level of reduction was obtained after monotherapy with light (500 J/cm^2^). When the *S. aureus* and *S. epidermidis* biofilms were exposed solely to light treatment (250 J/cm^2^), cell survival was reduced by 40 and 60%, respectively. However, the addition of gentamycin reduced biofilm culture counts by a further 20 and 15%, respectively, from their values in biofilms receiving aPDI alone (Barra et al., 2015). The influence of aPDI and an antibiotic on MRSA and methicillin-sensitive *S. aureus* (MSSA) biofilm cultures was described by Zhang et al. (2017), who used 5-ALA during photochemical reactions. Irradiation with a light dose of 360 J/cm^2^ and a subsequent 2 h incubation with 5-ALA (10 mM) gave an ~2 log_10_ reduction in the viable count of biofilm cells (Zhang et al., 2017). However, the effectiveness of this combined treatment was strain dependent. When antibiotics were present at very high concentrations (10x MIC), less biofilm was observed when aPDI was used with netilmicin, vancomycin and cefaclor for 7, 8 and 5 of 15 biofilm cultures, respectively (Zhang et al., 2017). The highest reduction in viable cells as a result of combined treatment, in comparison to monotherapy with light, was an ~2 log_10_ reduction in viable counts for biofilms (Zhang et al., 2017).

#### Exogenously administered porphyrin-based PS

The results presented in section *In vitro* studies: planktonic cultures: Exogenously administered porphyrin-based PS demonstrated a synergistic interaction between DP-aPDI and oxacillin for planktonic *S. aureus* cultures, which was also shown for biofilms (Iluz et al., 2018). Experiments conducted under shear flow conditions demonstrated that irradiation with a light dose of 15 J/cm^2^ and treatment with 17 μM deuteroporphyrin in the presence of oxacillin (1 μM) reduced the biofilm mass significantly more than treatment of biofilms with only oxacillin or DP-aPDI (Iluz et al., 2018). The most efficient reduction in viability came from a combination of aPDI and antibiotic applied to a MRSA biofilm (4 log_10_ greater than biofilms treated solely with light and DP). The reduction in viable cells in biofilms of MSSA or the heterogeneous vancomycin-intermediate *S. aureus* (h-VISA) receiving this treatment was ~2 log_10_ greater than in biofilms treated with only aPDI (Iluz et al., 2018). In 2009, Di Poto et al. (2009) as a first reported that aPDI combined with TMP and antibiotics exhibited increased effects against biofilms in *S. aureus* cultures. Combining the irradiation of biofilms with light doses ranging from 150 to 200 J/cm^2^ and the administration of 10 μM *meso*-tetrakis(n-methyl-4-pyridyl)porphine tetra tosylate (TMP), a PS, resulted in survival rates that were 30-70-fold lower than in untreated cultures. Moreover, when vancomycin was added after irradiation, the number of viable cells was reduced a further 5-fold from its value in samples treated only with aPDI. The vancomycin MIC value for biofilm cells not treated with aPDI was 10^3^-10^4^ higher than the MIC value after light treatment. The application of aPDI in combination with vancomycin gave a 5 log_10_ increased reduction in survival fraction over the reduction from independent treatments, which indicates the success of combining aPDI and antibiotic therapy (Di Poto et al., 2009).

#### Phenothiazines

A synergistic effect between aPDI and antibiotic therapy was also observed by Ronqui et al. (2016) when they used *E. coli* and *S. aureus* in both planktonic and biofilm cultures. The synergistic effect against planktonic cultures was determined for two different ciprofloxacin applications, one before and one after irradiation. In the case of biofilm cultures, the antibiotic was applied after aPDI treatment. The mode of aPDI and ciprofloxacin administration did not significantly affect the results in planktonic cultures. Irradiating *S. aureus* with a light dose of 2.8 J/cm^2^ in the presence of MB (50 μg/ml) and ciprofloxacin (0.5 μg/ml) resulted in a 5 log_10_ reduction in the viable cell count. In the case of *E. coli*, only a 1 log_10_ reduction was observed when the highest concentration of antibiotic was applied (50 μg/ml) after irradiation. When ciprofloxacin was administered before the aPDI treatment (2.8 J/cm^2^), the number of viable cells was reduced by 6 and 4 log_10_ for *S. aureus* and *E. coli*, respectively, but only when the highest concentration of PS was used (200 μg/ml). The order of application of antibiotic and aPDI significantly influenced the results only in case of *S. aureus*. In the case of biofilm cultures, a combined ciprofloxacin and aPDI treatment reduced the viable counts of *S. aureus* by 1 log_10_ more than treatment with only aPDI. For *E. coli* biofilms, the reduction of cell viability was 2.4 log_10_ greater than that in samples treated with an aPDI monotreatment. These results indicate the synergistic effect of the aPDI/ciprofloxacin combination against gram-positive and gram-negative microorganisms (Ronqui et al., 2016). The most effective biofilm inactivation resulted from a combination of aPDI and ciprofloxacin, which reduced viable cell counts for S. *aureus* by 1 log_10_ and for *E. coli* by 2.4 log_10_ more than monotherapy. The first published evidence stating the existence of a synergistic interaction between antimicrobials and aPDI was reported by Cassidy et al. (2012), who were focused on the *Burkholderia cepacia* complex, which is responsible for chronic cystic fibrosis pulmonary infections. For biofilm cultures, the assignment of synergy, antagonism and indifference to combined treatments was performed based on changes in a total viable count (synergy defined as a ≥2 log_10_ decrease in viable count; indifference <1 log_10_ change in viable count; antagonism defined as a ≥2 log_10_ increase in viable count). Planktonic cultures were also treated with light and PSs (TMP or MB). The aPDI monotherapy with MB resulted in a more than 3 log_10_ reduction for 4 of 6 tested *Burkholderia* strains (Cassidy et al., 2012). For biofilm cultures of *B. cenocepacia* (LMG 16659) and *B. multivorans* (LMG 18822), the highest reduction in viable counts was obtained when MB was used as a PS (5.09 and 4.53 log_10_, respectively). When only antibiotic was applied to biofilms, the bactericidal effect (a reduction by 3 log_10_) was determined for tobramycin in 5 of 6 tested strains and for meropenem, ciprofloxacin and piperacillin-tazobactam in 3 of 6 tested strains. For all strains and antibiotics (ceftazidime, chloramphenicol and ciprofloxacin), the combination of MB-aPDI and antibiotic reduced the viable counts more than antibiotic alone. Nevertheless, the synergistic effect was only observed for 3 isolates when a combined aPDI and chloramphenicol treatment was applied. The indifferent effect was dominant for treatments with a combination of aPDI and chemotherapeutical agents eradicating *B. cepacia* genomovars (Cassidy et al., 2012). The application of aPDI in combination with tobramycin or chloramphenicol increased the reduction in viable counts by ~4.5 log_10_ and 4 log_10_, respectively, over the reduction achieved by independent treatments. In 2017, Kashef et al. (2017) described the application of aPDI and linezolid to *S. aureus* biofilm cultures. For this purpose, TBO and MB were used as PS. Treatment of biofilms with only aPDI reduced the bacterial burden by no more than 0.6 log_10_ for MB and 0.7 log_10_ for TBO (Kashef et al., 2017). Similar effects were observed during exposure of *S. aureus* biofilms to only linezolid (0.7 log_10_ reduction). However, a combination of antibiotic and aPDI treatment increased the reduction in viable cell counts. When *S. aureus* strains were irradiated in the presence of TBO, the biofilm cell counts were reduced by 2.1–2.6 log_10_ by a preincubation with linezolid at a concentration of 1.6 mg/ml. A treatment combining irradiation of *S. aureus* biofilms with a light dose of 54.6 J/cm^2^, administration of MB and pretreatment with the same concentration of antibiotic reduced cell survival by 1.2 log_10_ (Kashef et al., 2017)_._ A combination of aPDI with linezolid and MB increased a reduction in survival fraction by 2 log_10_ over the reduction caused by monotherapy but only against biofilms of one *S. aureus* strain (UTMC 1440).

#### Rose bengal

Results by Perez-Laguna group presented in section Rose Bengal (RB) concerning the aPDI/gentamycin combined inactivation of *S. aureus* planktonic cultures were also confirmed for biofilms (Pérez-Laguna et al., 2018). The bactericidal effectiveness of light irradiation (18 J/cm^2^) and rose bengal (64 μg/ml) was lower than in case of planktonic cultures and reached 3.0 log_10_ reduction in viable counts. Nevertheless, the additional administration of gentamycin to aPDI treatment resulted in enhanced bactericidal effect finally leading to 6 log_10_ reduction in survival fraction. Combined aPDI and gentamycin treatment against *S. aureus* biofilm cultures was 2-fold more effective than aPDI monotherapy (Pérez-Laguna et al., 2018).

Table 2 summarizes the published results concerning the efficacy of combined aPDI/antimicrobial treatment *in vitro* for biofilm cultures.

**Table 2:** Summary of combined aPDI/antimicrobial treatments against biofilm cultures—*in vitro* experiments.

**References**	**Species**	**Photosensitizer/compound**	**Source of light**	**Wavelength [nm]**	**Intensity [mW/cm^2^]**	**Antibiotics**	**Max. viability reduction in comparison to monotherapy (light)**	**Max. viability reduction in comparison to monotherapy (antibiotic)**	**Applied methodology for determination of combined effect**
Krespi et al., 2011	*S. aureus* (MRSA)	–	Q-switched Nd-YAG SW laser, NIR diode laser	940	7894.7 (calculated)	Ciprofloxacin	47% (SW + NIR laser)	37%	Optical density measurement
Barra et al., 2015	*S. aureus S. epidermidis S. haemolyticus*	5-ALA	LED	630	No data	Gentamycin	20%	No data	Residual cell survival (CFU/ml)
							15%	No data	
							25%	No data	
Zhang et al., 2017	*S. aureus* (MRSA, MSSA)		LED	633 ± 10	No data	Netilmicin	~2 log_10_	No data	Bacterial viability (CFU/ml)
						Vancomycin	~2 log_10_	No data	
						Cefaclor	~1.5 log_10_	No data	
Iluz et al., 2018	*S. aureus* (MRSA, VISA, h-VISA)	DP	Blue light tube TL 20 W/03 ES	360-460	No data	Oxacillin	4 log_10_	–	Bacterial viability (CFU/ml)
							1.5 log_10_	5.5 log_10_	
							2 log_10_	–	
Ronqui et al., 2016	*S. aureus E. coli*	MB	Red LED	660	No data	Ciprofloxacin	1 log_10_	No data	Bacterial viability (CFU/ml)
							2.4 log_10_	No data	
Cassidy et al., 2012	*B. cepacia B. multivorans B. cenocepacia*		Paterson Lamp	635	200	Piperacillin-tazobactam	~2 log_10_ ~2.6 log_10_ –	~1 log_10_ ~2 log_10_ ~1.5 log_10_	Bacterial viability (CFU/ml)
						Meropenem	~2 log_10_ ~3 log_10_ ~0.9log_10_	1 log_10_ ~2 log_10_ ~3.1 log_10_	
						Ceftazidime	~1 log_10_ ~1.2 log_10_ ~0.4 log_10_	~2 log_10_ ~1.8 log_10_ ~2.3 log_10_	
						Tobramycin	3.5 log_10_ 4.5 log_10_ ~2.3 log_10_	~2 log_10_ 1.6 log_10_ ~0.9 log_10_	
						Chloramphenicol	~4 log_10_ ~3.1 log_10_ ~1.6 log_10_	~5 log_10_ ~3.8 log_10_ ~3.6 log_10_	
						Ciprofloxacin	~2 log_10_ ~2.8 log_10_ ~1.2 log_10_	~1.5 log_10_ ~3.3 log_10_ ~2.6 log_10_	
Kashef et al., 2017	*S. aureus*		Diode laser	660 630	91 26	Linezolid	~0.6 log_10_	~0.5 log_10_	Bacterial viability (CFU/ml)
Pérez-Laguna et al., 2018	*S. aureus*	RB	Green LED-lamp	515 ± 10	5.8	Gentamycin	~3.5 log_10_	No data	Bacterial viability (CFU/cm^2^)
Kashef et al., 2017	*S. aureus*	TBO	Diode laser	660 630	91 26	Linezolid	~2 log_10_	~2 log_10_	Bacterial viability (CFU/ml)
Di Poto et al., 2009	*S. aureus*	TMP	Tungsten lamp	400-800	166	Vancomycin	5 log_10_	No data	Reduction of surviving fraction

### *In vivo* studies

#### Fullerene derivatives

Only a few published reports concern the combined use of aPDI and antimicrobials in *in vivo* experiments. On the other hand, many studies describe the bactericidal efficacy of light therapies, employing various biomaterials, *ex vivo* tissues and animal models (Dai et al., 2009). In 2010, Lu et al. (2010) described the use of a mouse model to evidence the synergistic effect between aPDI and antibiotic treatment. *In vitro* analysis confirmed the high bactericidal effectiveness of aPDI against the tested strains, *P. aeruginosa* and *Proteus mirabilis* (30 μM sensitizer, fullerene derivative BF6 and irradiation with a light dose of 10 J/cm^2^). This approach reduced bacterial viability by >6 log_10_ in *P. aeruginosa* and totally eradicated *Proteus mirabilis* when the concentration of PS was 100 μM. *In vivo* experiments with fullerene derivative (180 J/cm^2^) were performed using the *Proteus mirabilis* wound infection model. Application of aPDI increased animal survival by 82% from its value in untreated animals. In the *P. aeruginosa* wound infection model, the bacterial burden was reduced 95% as a result of using aPDI with BF6 and an irradiance dose of 180 J/cm^2^. Despite an effective reduction of the bacterial load, *P. aeruginosa* survived the treatment, and after 3 days, all mice died from sepsis. An antibiotic treatment was used to increase the bactericidal efficacy of aPDI. A combined treatment using tobramycin (6 mg/kg each day) with light irradiation resulted in the survival of 60% of the infected animals; in contrast, 8% of mice treated with only tobramycin survived. Moreover, the infected wound was clear after 10 days, and no bacterial load was detected, indicating total eradication (Lu et al., 2010). These results are excellent evidence that indicate that combining light and antimicrobials can augment efficacy in both *in vitro* and *in vivo* studies.

#### Phenothiazines

Another combined treatment used in *in vivo* experiments refers to a method presented in the section *In vitro* studies: planktonic cultures: Phenothiazines *in vitro* results for the eradication of *Mycobacterium fortuitum* (Shih and Huang, 2011). White rabbits were used as a model of mycobacterial keratitis, and contact lenses infected with *M. fortuitum* cells were applied to their eyes. Treatment with only amikacin (20 mg/ml amikacin; 4 doses a day/7 days) gave an ~1 log_10_ reduction in viable bacterial cells in corneas. However, 7 days of a combined treatment (light dose of 97.5 J/cm^2^, MB 0.5% and amikacin 20 mg/ml; 4 doses a day/7 days) increased the reduction in the number of *M. fortuitum* cells in corneas by a further 0.91 log_10_ over the reduction in a group treated with only monotherapy (Shih and Huang, 2011). The use of a non-mammalian *in vivo* model was described by Chibebe Junior et al. (2013), who employed larvae of the greater wax moth, *Galleria mellonella*, in their *in vivo* experiments. A bacterial inoculum containing *Enterococcus faecium* or *E. faecalis* was injected into larvae hemocoel, and the antibacterial agents were administered within 2 h post-inoculation. PS (MB) was applied 90 min after bacterial cell injection, and irradiation with non-coherent red light of different fluences (0.9-18 J/cm^2^) was performed 30 min after infected *G. mellonella* had been administered with PS. All infected larvae except those infected with vancomycin-resistant *E. faecium* survived as a result of aPDI application. This species was next used to evaluate the bactericidal efficacy of the sequential application of aPDI and antimicrobials. Applying aPDI in combination with antibiotic made the survival rate of *G. mellonella* larger than that of organisms receiving only aPDI or vancomycin treatment (Chibebe Junior et al., 2013).

On other hand, Tanaka et al. (2013) reported in 2012 that the combined use of aPDI and antibiotics had the opposite effect. A MRSA mouse arthritis model was used in these studies. Based on previous experiments conducted by co-authors, a group of infected mice treated simultaneously with aPDI (with MB) and antibiotics were expected to yield the best results. However, therapeutic efficacy was not enhanced when linezolid was used. Nevertheless, when vancomycin was administered, the infection was reduced in intensity after 5 and 7 days. Irradiation with a light dose of 50 J/cm^2^ in the presence of MB (100 μM) without the administration of antibiotic totally eradicated pathogens, and no regrowth occurred in the first day after the treatment. A combined treatment did not result in such a positive effect. Even at day 7 of the experiment, the infection and bacterial load were still observed at the infection site. The authors concluded that the failure of combined treatment could result from an inhibition of neutrophil infiltration that was driven by light and antibiotic exposure. The reduced level of inflammatory cytokines caused by antibiotic administration contributed to the inhibition of cytokines, which are present as a result of aPDI (Tanaka et al., 2013).

Table 3 summarizes the published results concerning the efficacy of combined aPDI/antimicrobial treatments for *in vivo* models.

**Table 3:** Summary of combined aPDI/antimicrobial treatment—*in vivo* experiments.

**References**	**Species**	**Model organism**	**Photosensitizer/compound**	**Source of light**	**Wavelength [nm]**	**Intensity [mW/cm^2^]**	**Antibiotics**	**Combined treatment outcome**	**Max. viability reduction in comparison to monotherapy(light)**	**Max. viability reduction in comparison to monotherapy (antibiotic)**	**Applied methodology for determination of combined effect**
Lu et al., 2010	*P. aeruginosa Proteus mirabilis*	Mouse *Mus musculus*	BF6	Non-coherent Lamp, white light bandpass filter	400-700	200	Tobramycin	60% survival of mice after combined treatment	60% higher survival of mice	40% higher survival of mice	Bioluminescence imaging
Shin and Huang, 2011	*M. fortuitum*	Rabbit *Oryctolagus cuniculus*	MB	AlGalnP visible laser	650	100	Amikacin	2.1 log_10_ reduction of bacterial cells after combined treatment	No data	~1 log_10_	Quantitative analysis of viable colonies -bacterial viability (CFU)
Chibebe Junior et al., 2013	*E. faecium*	*Galleria mellonella*		Non-coherent	660 ± 15	No data	Vancomycin	6-fold increasein the time of survival of the larvae	~2.5-fold increase in the time of survival of the larvae	~1.7-fold increase in the time of survival of the larvae	Larvae viability (% survival)
Tanaka et al., 2013	*S. aureus* (MRSA)	Mouse *Mus musculus*		Xenon light source	660 ± 15	100	Vancomycin linezolid	Approx. 45-fold and 25-fold greater bioluminescence intensity (RLU)	25-fold greater RLU signal	2-fold greater RLU signal	Bioluminescence imaging
									20- fold greater RLU signal	~1-fold greater RLU signal	

### Clinical application

#### Endogenously produced porphyrins

In the case of clinical applications, aPDI, especially with the administration of 5-ALA, has been widely described in the treatment of skin infections such as acne vulgaris or psoriasis (Maisch et al., 2004). Nevertheless, the clinical studies that refer to the addition of chemotherapeutic agents during light therapy have also been reported. In 2017, four cases of patients with different skin disorders caused by *Mycobacterium* species (*M. chelonae, M. gordonae, M. gilvum*, and *M. fortuitum*) were treated with 5-ALA aPDI and antibiotic. Skin lesions of these patients were impregnated with 20% 5-ALA and irradiated with one dose of red light (100 J/cm^2^). This procedure was repeated every 10 days for 3-5 sessions with a combination of antibiotics (e.g., clarithromycin, moxifloxacin hydrochloride, or amikacin). All of the patients did not present any signs of recurrence 3 months after with combined treatment (Sun et al., 2017). More evidence of the enhanced bactericidal efficacy of an aPDI and antibiotics combination was presented by one patient with multiple skin abscesses caused by *M. fortuitum*. The same light source mentioned above and 20% 5-ALA were applied to their left hand every 10 days for 4 sessions with antibiotic therapy (clarithromycin, rifampin, levofloxacin, and ethambutol hydrochloride), while the right hand received the combined treatment in only two sessions and only after treatment with antibiotics. After each session of treatment, the area of lesion had significantly decreased in the left hand, while the significant effectiveness of aPDI was observed for the right hand after the first application of aPDI. Skin abscesses caused by *M. fortuitum* were effectively healed during the combined treatment, and no adverse reaction was observed after 3 months (Gong et al., 2016). Next, the bactericidal effectiveness of combined therapy using 5-ALA and antibiotics (minocycline) in acne vulgaris treatment was presented by Xu et al. (2017) in 2017. Forty eight patients were treated with minocycline (100 mg/day for 4 weeks) and once a week lesions were irradiated with light dose of 120 J/cm^2^ after skin incubation with 5% 5-ALA. Second group of patients was administered only with minocycline 100 mg/day for 4 weeks. Eight weeks after the treatment the effectiveness of combined therapy was higher than in case of minocycline monotherapy reaching the 80% reduction of inflammatory lesions (the reduction of lesions in minocycline monotherapy reached 50%) (Xu et al., 2017).

Table 4 summarizes the published results concerning the efficacy of combined aPDI/antimicrobial treatments in clinical studies.

**Table 4:** Summary of combined aPDI/antimicrobial treatments—clinical applications.

**References**	**Case no**.	**Species**	**Photosensitizer/compound**	**Source of light**	**Wavelength [nm]**	**Intensity [mW/cm^2^]**	**Dose of light-(number of sessions)**	**Antibiotics**	**Result of combined treatment**
Gong et al., 2016	1.	*M. fortuitum*	5-ALA	Laser optical fiber, Red LED-IB	635,633	− 84	− 100 J/cm^2^- (2)	Clarithromycin, rifampin, levofloxacin, ethambutol hydrochloride	Cure
Sun et al., 2017	2.	*M. chelonae*		Red LED-IB	633	84	100 J/cm^2^- (5)	Clarithromycin, moxifloxacin, amikacin, imipenem cilastatin sodium	Cure
	3.	*M. gordonae*					100 J/cm^2^- (3)	Clarithromycin, moxifloxacin, amikacin, sulfamethoxazole	Cure
	4.	*M. gilvum*					100 J/cm^2^- (3)	Moxifloxacin, clarithromycin	Cure
	5.	*M. fortuitum*					100 J/cm^2^- (4)	Amikacin, moxifloxacin, clarithromycin, rifampicin, ethambutol hydrochloride, levofloxacin	Cure
Xu et al., 2017	6.	*P. acnes*		Red Laser emitted diode LED	633	20-100	120 J/cm^2^- (4)	Minocycline	–

## Mechanisms underlying combined aPDI/antimicrobials treatment

**Figure 1:**
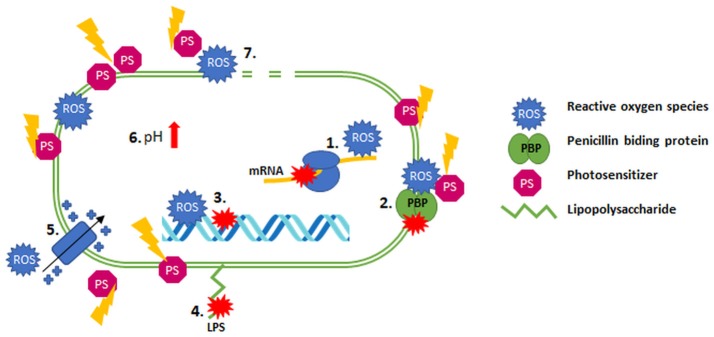
Possible mechanisms of synergy between aPDI and antibiotics. (1) mRNA inhibition; (2) dysfunction PBP; (3) DNA damage/inhibition of synthesis; (4) modification of LPS; (5) depolarization of the membrane; (6) alkalization of the cytoplasm; (7) permeabilization of the membrane.

The synergistic effects are often spectacular and indicate a high reduction in the MIC for microorganisms that had earlier manifested a significant level of resistance to an antibiotic (Fila et al., 2016). This phenomenon might result from the increased permeability of the cell envelope as a result of photoinactivation inducing its damage, which leads to greater antibiotic penetration into bacterial cells (Dai, 2017). Moreover, the enhanced bactericidal effect of antimicrobials in response to aPDI treatment might have been explained in the case of biofilm cultures by their disruption by different sources of light (e.g., shockwave laser), which could potentiate the action of antimicrobial agents (Krespi et al., 2011; Dai, 2017). Another possible mechanism underlying the synergistic effect of aPDI/antibiotic combinations is the oxidative stress that results from photochemical reactions inhibiting the expression of genes that are responsible for the antibiotic resistance; this mechanism was presented in research into a colistin-resistant *A. baumannii* strain (Boluki et al., 2017). The presence of these genes in other microorganisms (e.g., *K. pneumoniae, E. coli*, and *P. aeruginosa*) was also reported; this presence can explain the synergistic cooperation described for other bacterial species. The *mcr-1* gene is responsible for the modification of lipid A (phosphoethanoloamine), which leads to increased resistance to colistin, but this reaction can be reversed when the expression of this gene is inhibited (Boluki et al., 2017; Liu et al., 2017). The aPDI method probably leads to downregulated expression of these genes and the consequent reduced colistin resistance (Boluki et al., 2017). Possible explanations for aPDI/antimicrobial synergy include the ability of singlet oxygen and hydroxyl radicals to influence cellular homeostasis, the synthesis of nucleic acids (DNA and RNA), the alkalization of the cytoplasm and even the depolarization of the membrane (Pereira et al., 2017b). ROS can potentiate killing when antimicrobial agents such as ciprofloxacin, gentamycin, and fluoroquinolones are used (reported by the Brynildsen group in 2013; Brynildsen et al., 2013). In addition, the lower pH level in *Mycobacterium smegmatis* cells contributed to the increased sensitivity of bacterial cells to antibiotic treatment (Bartek et al., 2016). However, the possible connection between the production of ROS and increased pH levels is unexplained. The higher efficiencies resulting from combined treatments can be further explained by the hypothesis that PSs (e.g., MB) at very high concentrations can be substrates for efflux pumps, which might result in a competition between PSs and antimicrobial agents that increases the uptake of antibiotic by bacterial cells after the permeabilization of their membrane (Shih and Huang, 2011). Another possible explanation for the synergy of the combined treatment originates from the ROS production that occurs as a result of both aPDI and antibiotic treatments. ROS are involved in an alternative mechanism of action of numerous antimicrobials (Van Acker and Coenye, 2017). The aPDI method could thus simply potentiate the oxidative stress induced by antibiotic administration, leading to enhanced bactericidal effects and synergy. However, the mediation of the production and importance of the production of ROS by antibiotic action has been the subject of many disputes in the literature. Many studies report the production of ROS as a mechanism employed by antibiotics (Van Acker and Coenye, 2017), but contradictory data supports the lack of ROS-related mechanisms of antibiotic action in these cases (Liu and Imlay, 2013). Another possible mechanism involves the bactericidal effectiveness of aPDI toward persistent cells. Persistent microorganisms survive lethal effects of antibiotic treatments as a result of reversible and temporary phenotypic alterations (Cohen et al., 2013; Oppezzo and Forte Giacobone, 2017). This fact should be especially considered in *in vivo* studies because the presence of persistent cells can decrease the ratio of aPDI treatment effectiveness (recurrence of infection and bacterial growth). The fact that aPDI decreased the level of persistent cells could explain the higher efficiency of antimicrobial action. Exposure of bacterial cells to white, blue or red light clearly may significantly influence their susceptibility to antibiotics. This idea may be further supported by the presence of growth factors during pathogen incubation. For example, the concentration of iron in a culture medium or the temperature of incubation can significantly influence results. This influence was demonstrated by experiments performed by Ramírez et al. (2015) in 2015 and should be considered significant during synergy testing. The possible mechanisms underlying the synergistic effects of aPDI and antimicrobial agents are visualized in Figure 1.

The different aspects and factors described above are probable explanations of why antimicrobial agents work more efficiently when combined with aPDI, which was evidenced many times in the literature and discussed in this paper. The development of the alternative approach of combining aPDI and antibiotics therefore seems to be justified and desired. The combined treatment leads to not only the increased effectiveness of aPDI and antibiotics but also the decreased dosage of these chemotherapeutical agents, which may greatly slow the increasing rate of drug resistance (Dai, 2017).

## Conclusions

It is worth to underline that some of the papers described within the current review were just aimed at determination if combined treatments exert enhanced antibacterial outcome, without following the standard methodology to evaluate the synergistic effect, but in most of them (18 out of 27) authors of the cited papers indicated the existence of synergy between described antibacterial approaches. Most of the reported studies describing the combined aPDI/antibiotic treatment did not comply with the imposed standards for scientific literature that aim at analyzing the synergistic interactions between different biocidal approaches. The determination of synergistic interactions, which is especially desirable in the case of antibiotics and aPDI, will be possible only when the research is consistent with the existing guidelines. Following these guidelines may also be very helpful when comparing results obtained by different scientific groups and useful in defining reliable conclusions. We also emphasize here that a gold standard for the study of procedures involving light therapy and antibiotic interactions is lacking, thus comparing results obtained during aPDI by different scientific groups is very difficult. To facilitate adequate comparisons of results, we thus believe that antimicrobial susceptibility testing (AST) (even when combined with aPDI) should be performed in accordance with EUCAST or Clinical and Laboratory Standards Institute protocols. The employment of various antimicrobials exhibiting different mechanisms of action and aiming at various cellular targets is significant for synergy testing.

In general, the increase in bacterial inactivation was observed when both therapies were used in combination. Moreover, it is significant to indicate that beside the increase in bacterial inactivation with the combined therapy, the potential reduction in treatment time or/and in reduction in bacterial resistance development to antibiotics can be also expected when the combined therapy is used due to the reduced use of antimicrobials employed in the treatment.

One could expect that taking into consideration the described within this review paper published works it should be possible to draw constructive conclusions. Unfortunately, the lack of unified research methodology conducted in accordance with the available standards makes it impossible to reliably compare the results of the work obtained by various research groups. Looking for a combined/synergistic effect of various antibiotics (in accordance with applicable standards), the majority of experimental conditions are clearly defined, e.g., what species of bacteria is to be used, which strain that is characterized with the appropriate drug resistance profile should be employed, what antibiotic concentrations justify the inference about the increased bacterial effect of combined therapy etc. In the case of research on aPDI/antimicrobials combined treatment, the above mentioned parameters are set based on researchers' assumptions and experience. One can freely choose (i) a set of species and strains of microorganisms, regardless of the profile of their drug resistance; (ii) antibiotics and their concentrations; (iii) culture conditions, i.e., media, time and temperature of incubation; (iv) bacterial inoculum etc., which makes it difficult to draw constructive conclusions. In general, it is obvious that the degree of microbial inactivation in combined aPDI/antimicrobials treatment is significantly improved in accordance to monotherapies. Nevertheless, it is worth noting that in the case of some studies, such an enhanced effect was noted for concentrations of antibiotics equal to 10xMIC (Zhang et al., 2017) or 100xMIC (Di Poto et al., 2009), and in other works the same effect was obtained for sub-MIC concentrations equal to ½ or ¼ × MIC (Ronqui et al., 2016). It makes significant difference. Some studies present the effect of increased inactivation using wild-type (Fila et al., 2016; Kashef et al., 2017), antibiotic susceptible microbial strains (Branco et al., 2018), in other works this applies to multi-drug resistant isolates (Fila et al., 2016; Boluki et al., 2017; Iluz et al., 2018). The same problems can be identified when determining the conditions of aPDI. They largely stem from the experience and assumptions of the researchers. We hope that the indication of the above problems will convince research groups involved in a combined aPDI/antimicrobials treatment with the necessity to apply a unified research methodology based on available AST standards.

**Figure 2:**
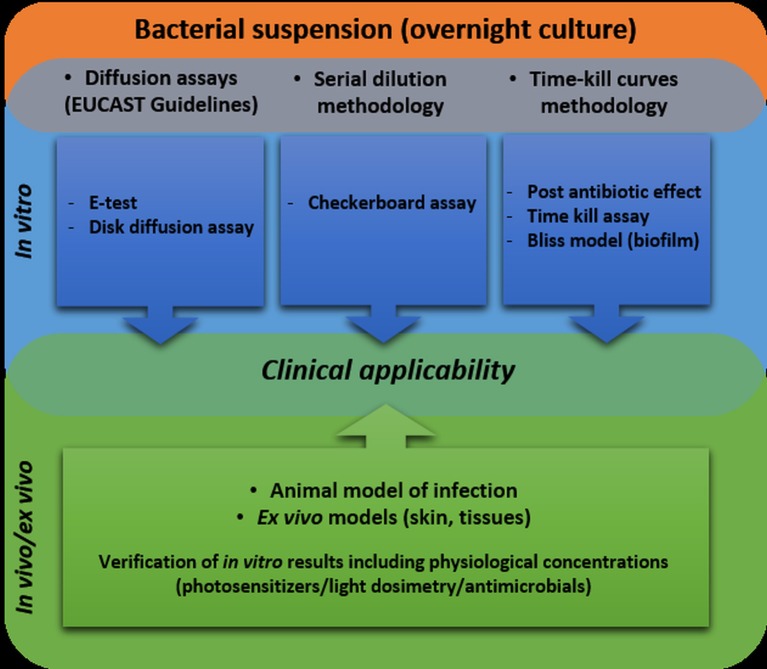
Workflow including recommended methodologies for testing the synergy between antimicrobial photodynamic inactivation and antibiotics.

Being aware of the existing issues, we created a workflow that shows the appropriate methodologies for synergy testing (Figure 2). An ideal approach would be an attempt to use as many as possible *in vitro* as well as *in vivo/ex vivo* tests to assess the synergistic interaction between tested antimicrobial approaches. We are convinced that only synergistic interactions that are confirmed in the maximum number of tests have a chance to be confirmed in clinical applications. In our studies, we repeatedly faced the problem that both various antibiotics and photosensitizers could reveal synergistic interactions when studied with some tests and simultaneously, other assays indicated the lack of such interaction. Therefore, our proposal is to use the largest possible number of *in vitro* tests before going into trials in *in vivo* and clinical applications. More importantly, we strongly believe that having a system of proposed methods will improve the research linking the problem of MDR and the clinical applicability of photodynamic inactivation.

In the current paper, we attempted to raise awareness of a problem and note the possible experimental approaches that will bring us closer to a final verification of which antimicrobials interact synergistically with aPDI and finally lead to enhanced bactericidal effectiveness.

## Author contributions

AW wrote the draft of the manuscript. MG has been involved in the coordination, conception, and design of the study and helped drafting manuscript. All of the authors have read and approved the final manuscript.

### Conflict of interest statement

The authors declare that the research was conducted in the absence of any commercial or financial relationships that could be construed as a potential conflict of interest.
